# MAUNet: a mixed attention U-net with spatial multi-dimensional convolution and contextual feature calibration for 3D brain tumor segmentation in multimodal MRI

**DOI:** 10.3389/fnins.2025.1682603

**Published:** 2025-10-08

**Authors:** Wenna Chen, Chuanqi Cai, Xinghua Tan, Rongfu Lv, Jincan Zhang, Ganqin Du

**Affiliations:** ^1^The First Affiliated Hospital, and College of Clinical Medicine of Henan University of Science and Technology, Luoyang, China; ^2^College of Information Engineering, Henan University of Science and Technology, Luoyang, China

**Keywords:** brain tumor segmentation, convolution, deep learning, multi scale, mixed attention

## Abstract

**Introduction:**

Brain tumors present a significant threat to human health, demanding accurate diagnostic and therapeutic strategies. Traditional manual analysis of medical imaging data is inefficient and prone to errors, especially considering the heterogeneous morphological characteristics of tumors. Therefore, to overcome these limitations, we propose MAUNet, a novel 3D brain tumor segmentation model based on U-Net.

**Methods:**

MAUNet incorporates a Spatial Convolution (SConv) module, a Contextual Feature Calibration (CFC) module, and a gating mechanism to address these challenges. First, the SConv module employs a Spatial Multi-Dimensional Weighted Attention (SMWA) mechanism to enhance feature representation across channel, height, width, and depth. Second, the CFC module constructs cascaded pyramid pooling layers to extract hierarchical contextual patterns, dynamically calibrating pixelcontext relationships by calculating feature similarities. Finally, to optimize feature fusion efficiency, a gating mechanism refines feature fusion in skip connections, emphasizing critical features while suppressing irrelevant ones.

**Results:**

Extensive experiments on the BraTS2019 and BraTS2020 datasets demonstrate the superiority of MAUNet, achieving average Dice scores of 84.5 and 83.8%, respectively. Ablation studies further validate the effectiveness of each proposed module, highlighting their contributions to improved segmentation accuracy. Our work provides a robust and efficient solution for automated brain tumor segmentation, offering significant potential for clinical applications.

## 1 Introduction

Brain tumors are major diseases that threaten human health and life. There are many brain tumor patients worldwide, and the number of patients is increasing every year (Zarenia et al., 2025).

As the most intricate organ in the human body, the brain demands extraordinary accuracy in tumor diagnosis and therapeutic interventions, owing to its highly complex anatomical organization and functional diversity. Brain neoplasms are broadly categorized into benign and malignant types. Although low-grade tumors, including specific gliomas and meningiomas, exhibit slow proliferation rates, they retain the potential to undergo malignant transformation. Conversely, malignant brain tumors like glioblastoma present a considerable threat to patient safety due to their highly invasive nature and rapid growth (Rehman et al., 2023; Zhou, 2023).

Magnetic Resonance Imaging (MRI) is essential for diagnosing and managing brain tumors, as it can precisely capture details about the internal composition of the tumors and structural changes in adjacent tissues. MRI employs multiple imaging sequences to elucidate multiple pathologic features of brain tumors. Furthermore, multimodal strategies provide complementary data by extracting features from multiple viewpoints, compared to single-modal techniques, thereby enhancing the representation of data and the differentiation capabilities of neural networks (Luo et al., 2021; Zhou et al., 2023).

However, with the dramatic increase in medical imaging data, traditional methods that rely on manual interpretation and tracking of brain tumors are increasingly shown to be inefficient and prone to error. Misdiagnosis is especially likely to occur when the same patient may have complex and diverse tumor presentations (Gencer and Gencer, 2025). This deficiency is further highlighted in such cases. To address this challenge, modern medicine is actively pursuing automated approaches, with significant emphasis on leveraging Artificial Intelligence (AI) capabilities (Ishaq et al., 2025). In recent years, Deep Learning (DL) has gained significant traction within the research community due to its notable advantages. A series of computer-aided diagnostic systems based on DL have been developed, which are used for image segmentation of various diseases, effectively enhancing diagnostic accuracy and efficiency (He et al., 2023; Cox et al., 2024).

In computer vision, Convolutional Neural Networks (CNNs) have demonstrated outstanding performance, especially for medical image analysis (Kshatri and Singh, 2023; Sharma et al., 2024; Budd et al., 2021; Sachdeva et al., 2024). The core of CNN is the convolutional operation. Convolutional layers comprise numerous convolutional filters that execute convolutions on incoming data utilizing a sliding window approach. In addition to normal convolutions, the broad category of CNNs encompasses many convolution processes, including dilated, depthwise separable, and group convolutions. These operations facilitate the improvement of feature extraction accuracy, minimize computational resource demands, or accomplish both goals concurrently. Dilated convolution, a particular variant of convolution operation in CNNs, contrasts with regular convolution in that the kernel examines the input image at a predetermined scale (Chen et al., 2018). In dilated convolutions, there is spacing between the convolution kernels, allowing them to skip certain pixels as they cover the image, thereby expanding the receptive field. Dilated convolutions enable the expansion of the receptive field without altering the image resolution, making it possible to extract more valuable information from the images.

Furthermore, attention mechanisms are regarded as a reliable method for enhancing visual tasks and have been extensively utilized in medical imaging (Chen Y. et al., 2023; Dutta et al., 2024; Shamshad et al., 2023; Sun et al., 2024). Attention mechanisms are generally divided into three primary types: self-attention mechanism, spatial attention mechanism, and channel attention mechanism. Among these, the channel-oriented variant emphasizes the significance of feature relevance across the channel axis in visual data. This type of attention mechanism allows networks to concentrate more on feature channels with higher information content, thereby enhancing the model’s representational capacity. The spatial attention mechanism emphasizes the significance of feature maps in the spatial dimension, identifying which portions of a picture are more pertinent for a particular activity. By amplifying the features of important areas while suppressing those of less importance, the network’s expressive power can be improved. Self-attention mechanism, also known as internal attention, is a core concept within the Transformer model. It enables the consideration of all positions within a sequence to compute the representation of that position, rather than processing information locally as convolutions do. The channel and spatial attention mechanisms are typically regarded as supplementary elements that augment feature representation within convolutional neural networks. Conversely, the self-attention mechanism serves as the core component of the Transformer architecture, capturing global dependencies through the recalibration of each element in the feature map.

Although U-Net-based medical image segmentation architectures have achieved notable progress, they still exhibit several inherent limitations. First, the predominantly local receptive field of standard convolutions insufficiently models both cross-channel and long-range spatial dependencies. For example, Zhou and Zhu (2023) limits the Transformer module to the bottleneck, whereas the encoder still stacks vanilla 3 × 3 × 3 convolutions, thereby precluding explicit modeling of global channel interactions. Similarly, Guan et al. (2022) proposed the AGSE-VNet model, in which the SE module only calculates first-order channel statistics without calibrating the spatial dimension. Second, multi-scale contextual fusion remains inadequate. Wang et al. (2023) and Peng and Sun (2023) adopted a single expansion rate or dual-scale convolution, but failed to generate dense receiving field coverage. Finally, skip-connection-based feature fusion tends to inject redundant information, diminishing feature discriminability. For instance, Li et al. (2022) directly connected encoder-decoder features of the same scale without gating or attention-based filtering, which might have impaired the accurate assessment of tumor cores and increased the false alarm rate. To overcome these challenges, we propose MAUNet, a 3D U-Net variant enhanced with mixed attention mechanisms corresponding to these three limitations. Moreover, Contemporary convolutional networks leverage the Squeeze-and-Excitation (SE) module and the Efficient Channel Attention (ECA) module, which primarily focuses on inter-channel interactions (Li et al., 2022; Hu et al., 2020). The Convolutional Block attention module (CBAM) improves feature representation through collaborative integration of channel and spatial dimension information (Wang et al., 2020). However, CBAM considers the overall relationship across the entire spatial domain. It is essential to recognize that contextual discrepancies during feature extraction may unintentionally incorporate extraneous information or inadequately deliver semantic indicators. Feature misalignment predominantly arises from iterative downsampling, resulting in spatial discordance between the output (such as features or predictions) and the input image (Woo et al., 2018a). In this paper, the MAUNet model is constructed, which addresses these issues by introducing spatial multi-dimensional convolution (SMDConv) and Context Feature Calibration (CFC). Our contributions are delineated as follows:

- The MAUNet architecture advances 3D brain tumor segmentation through innovative mixed-attention U-Net modifications.
- The core of SMDConv is “Spatial Multi-dimensional Weighted Attention” (SMWA). It compresses the four dimensions of the feature map - channel, width, height, and depth - into two numbers, “mean + standard deviation”, and then fuses them with a 1 × 1 × K small convolution. Finally, it dynamically recalibrates the weights of each dimension to enhance the feature representation.
- In the later stages, this architecture expands the receptive field through dilated convolutions and employs a CFC module that cascades self-attention pyramids to calibrate contextual features based on pixel-level contextual similarity.
- The features fused through skip connections are not directly sent into SMDConvs. Instead, they first undergo processing through a gated attention mechanism to enhance key features while suppressing non-essential ones.
- The empirical findings highlight the enhanced performance of our approach, while ablation studies validate the impact of key methodological choices.

## 2 Related work

Li et al. (2023) introduced the Fully Convolutional Network (FCN), which replaces the linear layers in the existing neural network architecture with convolutional layers for image segmentation. Although this model is efficient in semantic segmentation, its ability to recover spatial details is weak. The accuracy of the segmentation result is limited. Ronneberger et al. (2015) and Shelhamer et al. (2017) introduced the UNet architecture, a design that has seen widespread adoption within the field of medical image segmentation. This network’s encoder-decoder framework has established a robust groundwork for subsequent advancements in segmentation networks, acting as a dependable reference for ongoing research endeavors. Although this architecture performs well with a small amount of data, its effective receptive field is limited and its global context modeling ability is weak, making it prone to errors in large-sized targets or complex backgrounds. Xu et al. (2023) and Ronneberger et al. (2015) introduced PHCU-Net, a two-layer UNet architecture for melanoma segmentation. The model integrates contextual and detailed features through different pathways: global branches utilize a hierarchical attention mechanism. In contrast, local branches capture fine-grained patterns via a convolutional neural network. In addition, CBAM attention is introduced in jump connections to enhance the feature representation. Nevertheless, this architecture has a large number of parameters, high training costs, and a strong reliance on device resources. Qian et al. (2024) and Xu et al. (2023) enhanced the UNet structure by integrating comprehensive skip paths to facilitate the seamless fusion of multi-scale features representations within the decoding network. This approach efficiently collects detailed textures and extensive semantic content at all levels. It’s just that the computational cost is high. Furthermore, several scholars have introduced attention mechanisms to enhance the segmentation of brain MRI images, specifically targeting tumor areas (Qian et al., 2024). In their study on renal neoplasm analysis, Sitanaboina et al. (2023) and Vaswani et al. (2023) introduced an innovative 3D deep learning framework combining attention mechanisms with region-specific feature emphasis. The proposed architecture termed Attention 3D-CU-Net, enhances segmentation precision by dynamically weighting critical anatomical regions while suppressing less relevant data. This approach achieves superior tumor boundary delineation in renal imaging through adaptive spatial focusing within its convolutional blocks. However, the model has a large number of parameters, high consumption of training resources, and high requirements for the computing platform and video memory. Pereira et al. (2019) and Sitanaboina et al. (2023) proposed a new feature calibration module called SegSE for medical image segmentation based on SE, capable of spatially adaptive feature calibration while considering inter-channel relationships. This segmentation model does not perform very well, with an average Dice of only 80.9%. Chen S. H. et al. (2023) and Pereira et al. (2019) addressed kidney tumor segmentation by combining a Global Local Attention Network with a deepened UNet architecture, developing the GL-UNet11 model. However, due to the deepening of network layers and the dual attention mechanism, the training time and computational cost have significantly increased, resulting in a relatively low deployment efficiency.

Brain tumor segmentation research has increasingly focused on hybrid architectures merging U-net frameworks with attention mechanisms. Wang et al. (2023) and Qian et al., 2024 validated the performance of Transformer-based frameworks in this area with their TransBTS model. Meanwhile, Zhou and Zhu (2023) developed a framework incorporating uncertainty-aware attention fusion for enhanced segmentation accuracy. Their methodology commenced with a primary UNet generating initial tumor delineations. Subsequent analysis focused on probabilistic reliability assessment of these outputs through uncertainty quantification. These uncertainty-enhanced visualizations were combined with source data and processed through a secondary UNet architecture to refine the final segmentation. Although the model improves the segmentation accuracy through Bayesian uncertainty estimation and multi-attention fusion mechanism, its multi-stage training and multiple MC sampling significantly increase the computational cost and inference time. Wang et al. (2023) and Chen S. H. et al. (2023) developed GAM-Net, an innovative framework designed for brain tumor segmentation that leverages gradient information. This model features a Dual Convolutional Encoder (DCE) to capture more impactful characteristics from input data. By integrating a gradient pathway, GAM-Net adeptly exploits the encoded features through its DCE and introduces a Gradient-Oriented Decoding (GOD) mechanism. This GOD structure utilizes gradient information to improve the delineation precision of tumor margins in the brain. This architecture increases the occupation of video memory, consumes longer training time, and thus places higher demands on computing resources. Sobhaninia et al. (2023) and Wang et al. (2023) introduced a multi-scale cascaded multi-task framework. This model adopted a U-shaped architecture for its design. For the classification task, an integrated feature aggregation module was employed to boost the precision in distinguishing tumor types. Notably, their approach demonstrated outstanding results when evaluated on the Chen dataset. Although this framework performs well in segmentation and classification accuracy, it has a complex structure, a long training time (28 h), and relies on ROI pre-detection. The overall process is relatively heavy, which is not conducive to rapid deployment. Guan et al. (2022) introduced an innovative AGSE-VNet framework aimed at segmenting brain tumors. In this model, they incorporated SE components into the encoding stage and Attention-Guided (AG) filters within the decoding phase. The SE blocks were designed to amplify significant channel-wise features while diminishing less relevant ones through analyzing inter-channel dependencies, thus boosting segmentation precision. Meanwhile, the AG filters employed attention-based strategies to refine edge detection, effectively filtering out noise and extraneous data, which further refined the delineation accuracy. The performance of this model is not good, with an average Dice of 74% on the BraTS 2020 dataset. Li et al. (2022) and Sobhaninia et al. (2023) presented a CGA U-Net approach for brain tumor delineation, merging category-specific attention mechanisms with the original U-Net design. The Supervised Attention Module (SAM) forms the core of this framework, which leverages long-range dependencies in feature maps to achieve superior accuracy and computational stability without compromising efficiency. Furthermore, the researchers designed a novel intra-class pixel update mechanism. This technique improves feature representations by combining semantic cues from pixels with matching labels, consequently enhancing the model’s capacity to analyze contextual connections among same-class pixels. This model relies on a manually set number of categories. When the number of tumor categories increases, its generalization is limited. Peng and Sun (2023) introduced the AD-Net framework for brain tumor segmentation by leveraging dual-scale convolutional features to isolate channel-specific information and dynamically modulating weights via trainable parameters. They further incorporated a deep supervision mechanism to enhance model robustness. The average Dice of the three items in the BraTS20 validation set is 82%.

It is evident from the discussion that U-Net offers a practical architecture for segmenting medical images. Nonetheless, several considerations remain crucial for enhancement. Initially, employing elementary convolutional layers alone for feature extraction does not sufficiently emphasize the spatial and channel-specific characteristics of images. Moreover, depending exclusively on convolution operations tends to prioritize local details while overlooking broader contextual information. Lastly, directly transmitting integrated features to the decoder via skip connections may result in unnecessary duplication and diminished feature clarity. To tackle these challenges effectively, the MAUNet model has been introduced. The MAUNet model effectively enhances the multi-scale feature expression and global context modeling capabilities of three-dimensional brain tumors in multimodal MRI by introducing a spatial multi-dimensional weighted attention mechanism, a cascaded pyramid context feature calibration module, and gated jump connections. At the same time, the segmentation accuracy has been improved and redundant information has been suppressed. The proposed model solves the problems raised above.

## 3 Proposed method

Presented here is a novel U-architecture-based 3D brain tumor segmentation model, namely MAUNet, which integrates several attention mechanisms to improve segmentation accuracy. Subsequently, this section elaborates on the core framework of the model, its constituent elements, and the employed loss functions.

### 3.1 MAUNet structure

**FIGURE 1:**
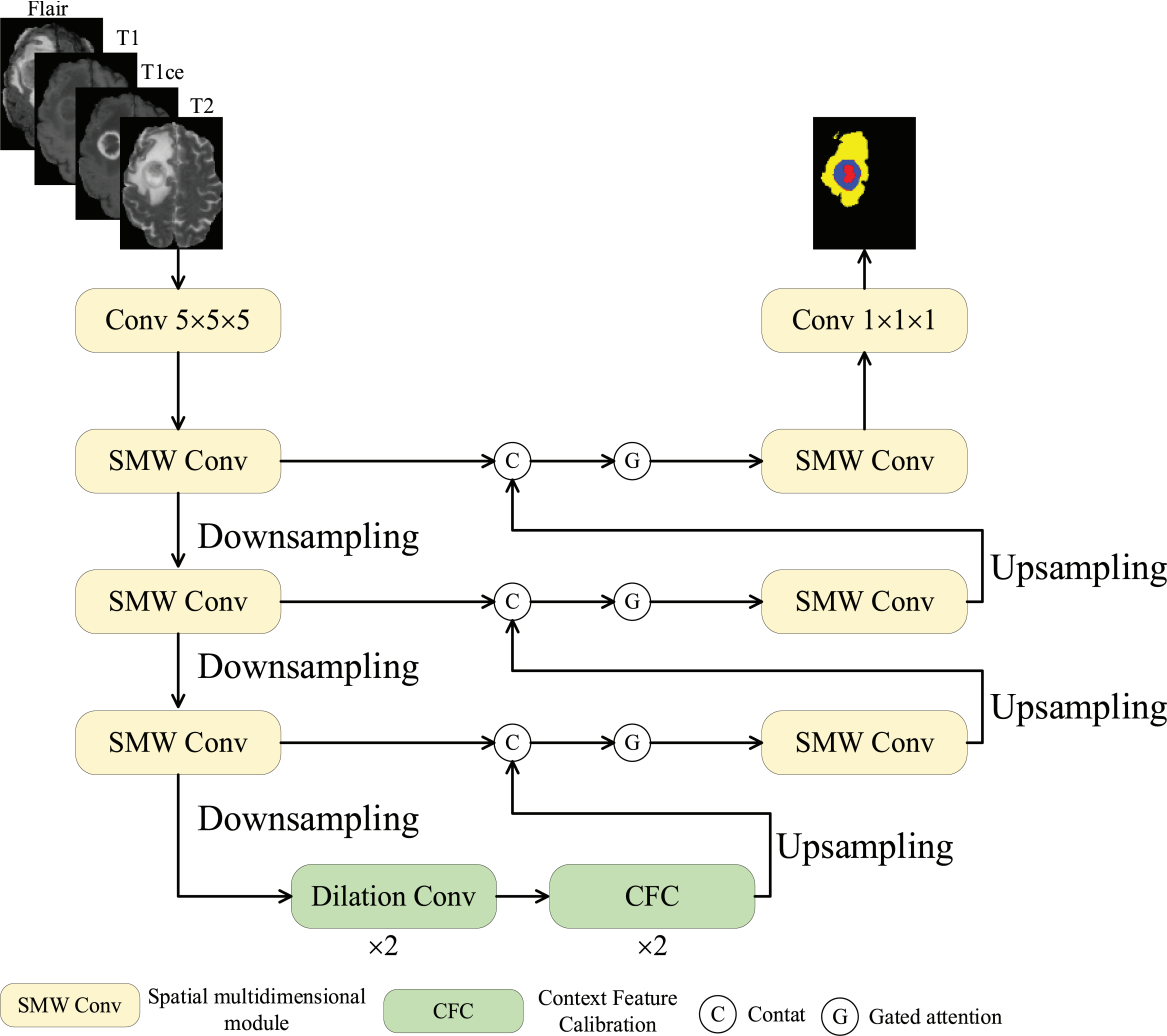
MAUNet structure.

Figure 1 depicts the structural design of MAUNet. The encoder of MAUNet primarily consists of three SMDConv modules and three downsampling layers, whereas the decoder comprises three SMD Conv modules and three upsampling layers. Each SMDConv module has two convolutional layers and a spatial multidimensional weighted attention (SMWA) module, the latter being the core part. MAUNet leverages four imaging modalities of brain tumors as input, processed via a convolutional module featuring a 5 × 5 filter and 32 feature maps. Within the encoder, each successive layer doubles its feature channels while employing a 2 × downsampling factor. At the MAUNet model’s base, two dilated convolution layers and two CFC modules exist. Dilated convolutions are frequently utilized in convolutional neural networks to augment the model’s receptive field. The CFC modules are employed to calculate the similarity between pixels and context, which strengthens the integration of contextual information in the model. Within the decoder stage, the skip-connected fused features undergo gated attention processing prior to being fed into the feature sampling module. The gating operation is performed by a sigmoid function, which maps any real-valued input to the open interval (0, 1). its output can be interpreted as a retention probability. In MAUNet, the sigmoid converts channel-wise scores produced by an MLP into weights in the range [0, 1]: values approaching 1 indicate that the corresponding channel–spatial features are important and should be preserved, whereas values approaching 0 lead to their suppression. Therefore, the gating mechanism softly selects features that propagate by skipping connections, allowing important information to pass through while reducing the passage of redundant information, rather than forcibly discarding them. By integrating a gated attention mechanism, the model dynamically prioritizes and focuses on critical regions within the input data, enhancing its ability to capture salient features. By learning the distribution of attention, the model acquires better generalization capabilities, performing well even on unseen data. Downsampling involves max-pooling techniques to condense the model, decrease the parameter count, and filter feature maps to preserve pertinent information, whereas upsampling applies trilinear interpolation.

### 3.2 Spatial multi-dimensional weighted attention

**FIGURE 2:**
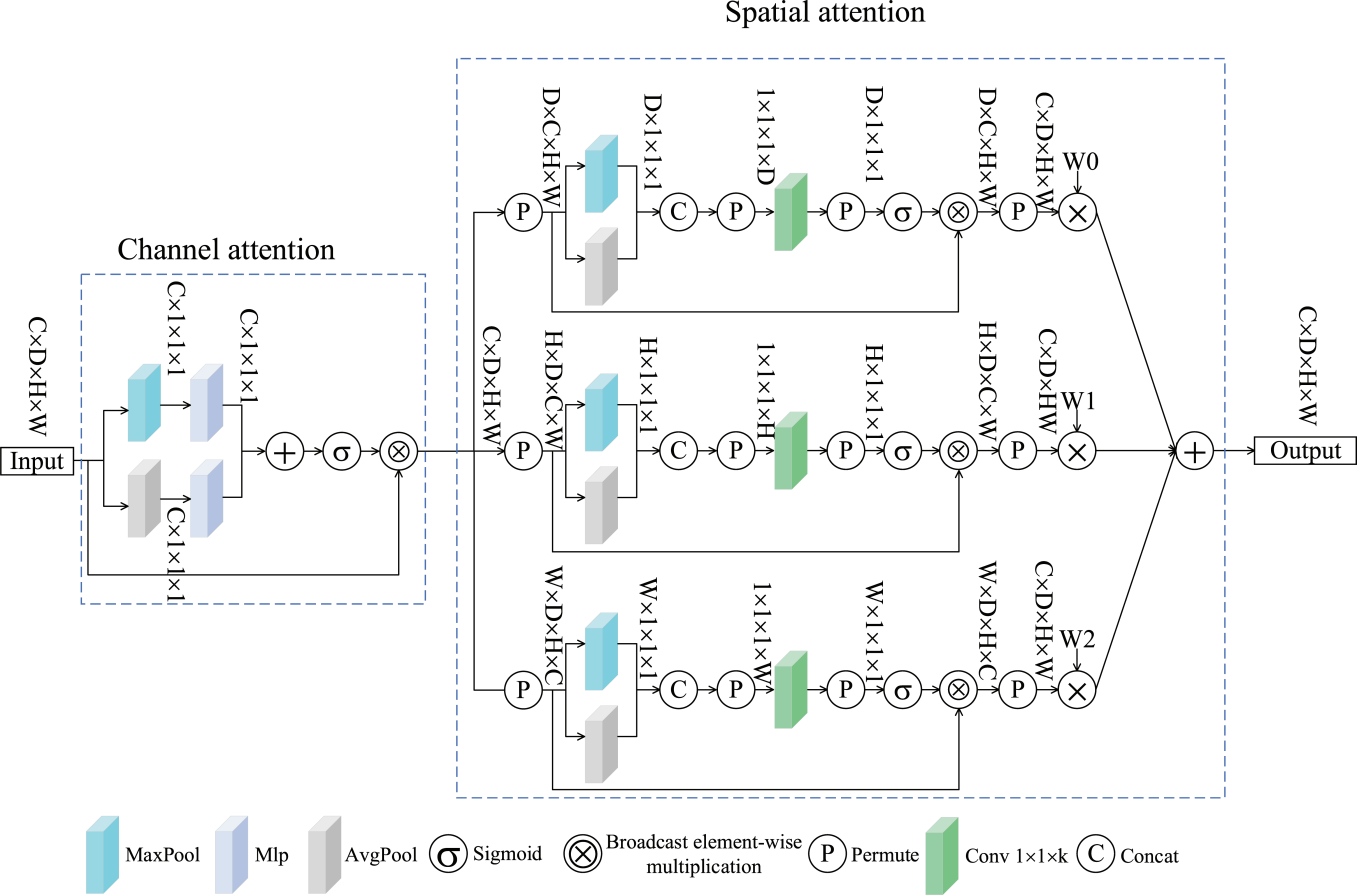
SMWA block.

Spatial multi-dimensional weighted attention was conceived based on (Woo et al., 2018b) and (Yu et al., 2023). Figure 2 depicts the SMWA architecture, which integrates two attention modules: channel-based weighting and spatial feature enhancement. The former assigns dynamic weights to feature channels, emphasizing their relative significance, while the latter refines spatial dependencies across the input data. Consequently, the processed features exhibit variations along the channel dimension, emphasizing important channels while suppressing less relevant ones. The input features undergo individual processing by both the channel attention mechanism and the spatial attention mechanism. This attention module for channels incorporates an average pooling layer, a maximum pooling layer, and a Multilayer Perceptron (MLP). Given an input feature *f*∈R^C×D×H×W^, where C represents the channel count, D the depth, H the height, and W the width of the spatial feature map, the processed features are presented through the channel attention mechanism, as shown in Equation 1.


(1) $$f_{c}=s\,i\,g\,m\,o\,i\,d\,\left(M\,L\,P\,\left(A\,v\,g\,p\,o\,o\,l\,\left(f\right)\right)+M\,L\,P\,\left(M\,a\,x\,P\,o\,o\,l\,\left(f\right)\right)\right)⊗f$$

here, the operator ⊗ signifies broadcast Hadamard product, while *f*_*c*_ corresponds to the output features from the channel attention mechanism, with *f*_*c*_∈R^C×D×H×W^. To derive compact spatial descriptors, dual pooling operations (average and max) are applied to input feature map *f*, producing *f*_*cavg*_∈R^C×1×1×1^ and *f*_*cmax*_∈R^C×1×1×1^. These compressed representations are concatenated and fed into a multilayer perceptron (MLP), after which a sigmoid activation normalizes the outputs to yield the final channel-wise attention coefficients weight_*c*_. Multiplying weight_*c*_ with the original feature *f* yields the channel-attended features *f*_*c*_, whereby different channels are assigned varying weights according to their importance.

After processing through channel attention, the features *f*_*c*_, are further optimized by the spatial single-dimensional weighted attention module, producing the features *f*_*s*_. Figure 2 illustrates the SMWA applies the same weighting method to the spatial dimensions D, H, and W. The compression stage of the spatial attention mechanism combines both max- and average-pooling operations, along with components C and P. The excitation component comprises convolutional layers, P, a sigmoid function, and element-wise multiplication through broadcasting. To illustrate the operation of the spatial attention module, consider the D-dimensional branch as an example. Firstly, *f*_*c*_ undergoes compression. The format of *f*_*c*_ is transformed, resulting in the feature *f*_*sd*_∈R^D×C×H×W^. This feature *f*_*sd*_ is then fed into both the max pooling and average pooling layers, yielding the compressed features *f*∈R^D×1×1×1^. Therefore, the input of the compressed part *f*_*c*_ can be expressed by Equation 2.


(2) $$f_{s\,d}^{\prime }=\left[A\,v\,g\,P\,o\,o\,l\,\left(p\,e\,r\,m\,u\,t\,e\,\left(f_{c}\right)\right),M\,a\,x\,P\,o\,o\,l\,\left(p\,e\,r\,m\,u\,t\,e\,\left(f_{c}\right)\right)\right]$$

In this case, the permute function represents the transformation of the feature data.

The compressed feature, denoted as *f*, is subsequently fed into the excitation component. Initially, *f* undergoes a data format transformation to produce *f” sd*. Subsequently, *f” sd* traverses a convolutional layer where, unlike standard designs, the dimensions of the convolutional kernel are designated as (1, 1, k), as demonstrated in Equation 3.


(3) $$f_{s\,d}^{\prime \prime \prime }=\overset{K}{\underset{x=1}{\sum }}W\,\left(x\right)\times p\,e\,r\,m\,u\,t\,e\,\left(f_{s\,d}^{\prime }\,\left(x\right)\right)$$

The spatial features of the D branch, after passing through the spatial attention module, yield an output that belongs to the space *f*_*sdout*_∈R^C×D×H×W^. The above formulation can be expressed by Equation 4.


(4) $$f_{s\,d\,o\,u\,t}=p\,e\,r\,m\,u\,t\,e\,\left(s\,i\,g\,m\,o\,i\,d\,\left(p\,e\,r\,m\,u\,t\,e\,\left(f_{s\,d}^{\prime \prime \prime }\right)\right)⊗p\,e\,r\,m\,u\,t\,e\,\left(f_{c}\right)\right)\times W_{0}$$

Similarly, the output characteristics of the H and W branches can be calculated using Equations 5 and 6.


(5) $$f_{s\,h\,o\,u\,t}=p\,e\,r\,m\,u\,t\,e\,\left(s\,i\,g\,m\,o\,i\,d\,\left(p\,e\,r\,m\,u\,t\,e\,\left(f_{s\,h}^{\prime \prime \prime }\right)\right)⊗p\,e\,r\,m\,u\,t\,e\,\left(f_{c}\right)\right)\times W_{1}$$

(6) $$f_{s\,w\,o\,u\,t}=p\,e\,r\,m\,u\,t\,e\,\left(s\,i\,g\,m\,o\,i\,d\,\left(p\,e\,r\,m\,u\,t\,e\,\left(f_{s\,w}^{\prime \prime \prime }\right)\right)⊗p\,e\,r\,m\,u\,t\,e\,\left(f_{c}\right)\right)\times W_{2}$$

here, W0, W1 and W2 represent learnable parameters designed to dynamically optimize weighting for spatial features across dimensions D, H and W. Ultimately, the automatic weighting fusion within the spatial attention mechanism generates the features as shown in Equation 7.


(7) $$f_{s}=f_{s\,d\,o\,u\,t}+f_{s\,h\,o\,u\,t}+f_{s\,w\,o\,u\,t}$$

### 3.3 Context feature calibration

**FIGURE 3:**
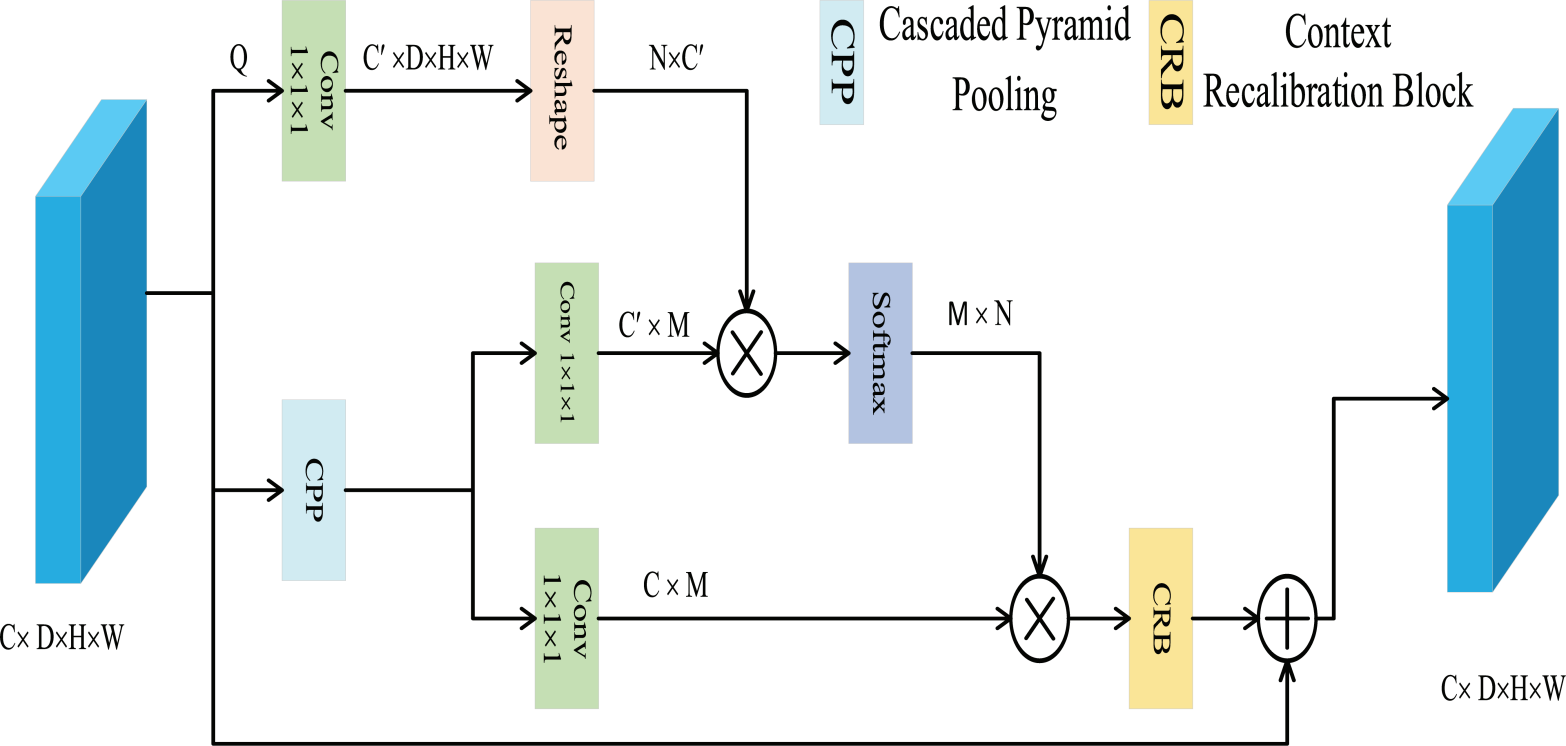
The structure of CFC.

The CFC approach is utilized to resolve the problem of pixel context mismatch (Woo et al., 2018a), as depicted in Figure 3. CFC employs a cascaded pyramid pooling structure, an effective technique to capture multi-scale contextual features within visual data. This hierarchical configuration ensures that pooling outputs across diverse resolutions are iteratively integrated, enabling the extraction of comprehensive contextual representations with reduced computational overhead. Through this architecture, the model gains the ability to interpret both localized and holistic contextual dependencies at varying granularities–a critical factor for distinguishing nuanced boundaries and intricate details among distinct categories.

The CFC module efficiently captures multi-scale contextual information through the Cascaded Pyramid Pooling (CPP) block, which is implemented using three parallel max-pooling layers. Given a feature map *x*ϵ∈R^C×D×H×W^, it is first reduced to *Q*∈R^C^′^×D×H×W^ convolutional layer, where C’ is set to be less than the original number of channels C. Subsequently, the CPP block extracts multi-scale contexts Z∈R^C×M^ from Q, with M representing the total number of output features across all scales. Following this, Z is processed through two convolutional layers to generate two forms of contextual representations K and V, belonging to R^C^′^×M^ and R^C×M^, respectively. Then, we employ cosine similarity to measure the similarity between each pixel feature vector and its contextual features. This metric focuses solely on vector directional consistency within the normalized feature space, thereby effectively capturing semantic associations. Its output ranges from [−1, 1], providing numerically stable input to subsequent softmax layers and ensuring the reliability of weight distributions. It calculates the affinity *A*∈R^N×W^ between the pixel and the context through matrix multiplication and the softmax function, where *N* = D × H × W, and the affinity between the i-th pixel and the j-th context is represented by *A*_*i, j*_, which is calculated according to the Equation 8.


(8) Ai,j=exp⁡(Qi⋅Kj)∑j=′1Mexp⁡(Qi⋅Kj′)

Finally, matrix multiplication is executed with V and the transpose of A, represented as A*^t^*, to derive the calibrated semantic context E∈R^C×N^. This is then reshaped back to its original dimensions of R^C×D×H×W^. Subsequently, E undergoes further refinement through the Context Recalibration (CRB), resulting in the generation of E’, defined by the following Equation 9.


(9) E=′tanh(W2(W1(X+E)))⋅E+E

In this process, W1 and W2 represent the utilized 1 × 1 × 1 and 3 × 3 × 3 convolutional layers, respectively. The tanh function is employed to eliminate redundant information and highlight beneficial details within the context. Finally, an element-wise addition is conducted on X and E’ to produce the final result *Y*∈R^C×D×H×W^, which is shown in Equation 10.


(10) Y=X+E′

### 3.4 Loss function

In DL, loss metrics serve as fundamental components that evaluate prediction accuracy by measuring deviations between estimated and true outcomes. These metrics numerically represent error magnitudes, guiding neural networks to refine their performance during training. A core objective of the learning phase involves iterative parameter optimization to reduce the loss metric, thereby enhancing alignment with training data. The backpropagation mechanism computes layer-wise gradients of these errors, enabling systematic error reduction. Subsequently, optimization algorithms employ gradient-based methods to iteratively update network parameters, driving the model toward optimal performance.

To improve the model’s predictive accuracy, a combined loss function that integrates Binary Cross Entropy (BCE) and Dice loss is adopted, as shown in Equation 11:


(11) $$l_{l\,o\,s\,s}=\alpha \times l_{b\,c\,e}+\beta \times l_{d\,i\,c\,e}$$

here, α and β are hyperparameters indicating the weights of *l*_*bce*_ and *l*_*dice*_, which are set to 1 and 0.5, respectively.

In medical imaging applications such as tumor delineation, class imbalance frequently arises due to the limited spatial occupancy of pathological structures relative to surrounding tissues. This imbalance manifests as a pronounced disproportion between foreground regions (e.g., lesions) and background pixels, complicating model optimization (Sudre et al., 2017). To tackle this issue, the Dice loss function, which is based on the Sørensen-Dice coefficient, is extensively used in segmentation tasks. Unlike cross-entropy, which operates on pixel-wise probability distributions, this loss quantifies spatial overlap between model predictions and ground truth annotations, thereby prioritizing underrepresented target areas. Specifically, the coefficient evaluates congruence across two sets: the algorithm-generated segmentation mask and expert-annotated reference data. By emphasizing region-based similarity over pixel-level accuracy, the Dice loss mitigates bias toward dominant classes while enhancing sensitivity to subtle anatomical features. Equation 12 displays its mathematical expression.


(12) $$l_{d\,i\,c\,e}=1-\frac{2\times \left|A\cap B\right|}{\left|A\right|+\left|B\right|}$$

here, A represents the ground truth brain tumor region, while B corresponds to the predicted region.

## 4 Results and discussion

### 4.1 Datasets and parameter settings

This study utilizes two publicly accessible and reputable brain tumor segmentation datasets, BraTS2019 and BraTS2020 (Bakas et al., 2017, 2019; Menze et al., 2015), to assess the proposed model. As a global initiative, the Brain Tumor Segmentation Challenge (BraTS) focuses on advancing automated glioma segmentation through curated datasets. This competition accelerates the research and development of segmentation algorithms by providing standardized resources for benchmarking. These datasets include multimodal MRI scans from patients, specifically T1, T1ce, T2, and FLAIR images. All images have undergone standardization and resizing to a consistent dimension of 155 × 240 × 240, which aids in algorithm development and comparison. In addition, each instance has a segmentation mask manually annotated by a domain expert to identify several tumor regions. In this study, the original brain tumor images sized at 155 × 240 × 240 were resized to the standard dimension of 160 × 240 × 240. Subsequently, these images were further segmented into five smaller segments, each with a size of 32 × 128 × 128. Before segmentation, all images were normalized to reduce the effects of variations in grayscale values and improve the stability of model training. Random flipping was applied during the training phase as a data augmentation technique to lower the likelihood of overfitting, thereby enhancing the model’s ability to generalize.

The model was implemented in PyTorch 1.13 and evaluated on Ubuntu 20.04 with Python 3.9. For hardware configuration, we employed an RTX 4070 GPU with 12 GB of VRAM and an Intel i5-13400F processor for computational tasks. Training was conducted using the Adam optimizer, initialized with a learning rate of 0.0001. The random seed set during segmentation is 41. Additionally, early stopping was implemented to avert overfitting. The specific parameter configurations employed in the trials are outlined in Table 1.

**TABLE 1:** The parameter settings of the network.

Parameters	Value
Initial learning rate	0.0001
Batch size	1
Optimizer	Adam
Epoch	100
Random seed	41

### 4.2 Computational efficiency and deployment analysis

To further assess the practical applicability of the proposed method, Table 2 summarizes the GPU memory footprint, total training time, and inference latency achieved on both the BraTS 2019 and BraTS 2020 datasets. All experiments were conducted on an identical workstation.

**TABLE 2:** The computational efficiency metrics of the proposed model on the BraTs2019 and BraTs2020 datasets.

Dataset	GPU-AvgM (GB)	Train time (min)	Inference time (min)	Average dice (%)
BraTs2019	9.2	420.12	7.96	84.5
BraTs2020	9.1	700.48	10.48	83.8

As shown in Table 2, on the BraTS2019 dataset, the proposed model consumed 9.2 GB of GPU memory, completed training in 420.12 min, performed inference in 7.96 min, and achieved an average Dice score of 84.5%. Similarly, on the BraTS2020 dataset, GPU memory usage remained 9.1 GB, training lasted 700.48 min, inference took 10.48 min, and the average Dice score was 83.8%. These results indicate that the proposed method achieves good segmentation accuracy while retaining computationally reasonable efficiency.

### 4.3 Evaluation metrics

In this study, we implemented a comprehensive set of metrics to evaluate the segmentation efficacy of the model, encompassing the Dice Similarity Coefficient (DSC), sensitivity (True Positive Rate, TPR), and specificity (True Negative Rate, TNR). The DSC served as the primary quantitative benchmark for segmentation accuracy, quantifying the overlap between predicted and ground-truth regions. Precision measures the accuracy of the model in predicting positive cases. Sensitivity reflects the model’s capability to accurately identify positive cases, whereas specificity assesses the model’s accuracy in identifying negative cases (Tripathi and Bag, 2023). The mathematical formulas for these evaluation criteria are shown in Equations 13–16.


(13) $$D\,i\,c\,e=\frac{2\,T\,P}{2\,T\,P+F\,P+F\,N}$$

(14) $$S\,e\,n\,s\,i\,t\,i\,v\,i\,t\,y=\frac{T\,P}{T\,P+F\,N}$$

(15) $$S\,p\,e\,c\,i\,f\,i\,c\,i\,t\,y=\frac{T\,N}{T\,P+F\,P}$$

(16) $$P\,r\,e\,c\,i\,s\,i\,o\,n=\frac{T\,P}{T\,P+F\,P}$$

here, TP (True Positives) indicates the count of correctly identified positive cases, while TN (True Negatives) represents the count of accurately recognized negative cases. FP (False Positives) refers to the count of negative cases that are misclassified as positive, and FN (False Negatives) is the count of positive cases that are wrongly classified as negative.

### 4.4 Hyperparameter optimization

To further validate the rationale behind the hyperparameters employed in the MAUNet model, this section presents a systematic experimental analysis of key hyperparameters, including the learning rate, optimizer, and random seed. The model was trained on the BraTS2019 training set, and its segmentation performance was evaluated on the test set, with the Dice coefficient serving as the primary evaluation metric.

As summarized in Table 3, the impact of different learning rates on model performance was first evaluated by testing values of 0.001, 0.0001, and 0.00001. The experimental results demonstrate that a learning rate of 0.0001 enabled the model to achieve Dice scores of 91.20, 84.00, and 78.40% for the whole tumor (WT), tumor core (TC), and enhancing tumor (ET) regions, respectively. This configuration yielded a mean Dice score of 84.53%, significantly outperforming other settings. Subsequently, a comparison was conducted among three prevalent optimizers: Adam, SGD, and RMSprop. The results reveal that the Adam optimizer yielded superior performance across all evaluation metrics. Finally, experiments were conducted under multiple random seed values (26, 41, 2023). The highest Dice score is achieved when the seed value is set to 41.

**TABLE 3:** Performance comparison of MAUNet models with different hyperparameters.

Parameters	Value	Dice (%)			
		WT	TC	ET	Average
Lr	0.001	88.82	79.06	75.90	81.26
	0.00001	89.47	79.76	76.67	81.97
	0.0001	91.20	84.00	78.40	84.53
Optimizer	RMSprop	89.16	79.85	76.39	81.80
	SGD	87.70	78.23	72.97	79.63
	Adam	91.20	84.00	78.40	84.53
Random seed	42	89.37	80.63	76.12	82.04
	2023	89.14	78.45	75.00	80.86
	41	91.20	84.00	78.40	84.53

In summary, the hyperparameter combination established in this study (learning rate: 0.0001, optimizer: Adam, random seed: 41) demonstrated optimal performance across the evaluated metrics and experimental conditions.

### 4.5 The segmentation results of the model

This section comprehensively evaluates the proposed model’s performance on the BraTS2019 and BraTS2020 test datasets. The model demonstrated outstanding performance on the BraTS2019 dataset, achieving Dice scores of 91.2% for the whole tumor (WT), 84.0% for the tumor core (TC), and 78.39% for the enhancing tumor (ET) regions. The corresponding values for sensitivity, specificity and precision were 91.6, 84.8 and 82.7%, 91.2, 85.6 and 77.6%, and 90.07, 86.98 and 79.23%, respectively. On the BraTS2020 dataset, the model demonstrated similarly strong performance, achieving Dice scores of 90.1, 84.2, and 77.2% for the WT, TC, and ET regions. The sensitivities were 90.6, 85.1, and 77.3%; the specificities were 90.6, 86.9, and 81.7%; and the precision values were 90.63, 86.94, and 81.70%, respectively. A comprehensive set of evaluation metrics can be found in Table 4.

**TABLE 4:** Segmentation results of the model on the test sets of BraTS2019 and BraTS2020.

Dataset	Dice (%)			Sensitivity (%)			Specificity (%)			Precision (%)		
	WT	TC	ET	WT	TC	ET	WT	TC	ET	WT	TC	ET
BraTs2019	91.2	84.0	78.4	91.6	84.8	82.7	91.2	85.6	77.6	90.07	86.98	79.23
BraTs2020	90.1	84.2	77.2	90.6	85.1	77.3	90.6	86.9	81.7	90.63	86.94	81.70

**FIGURE 4:**
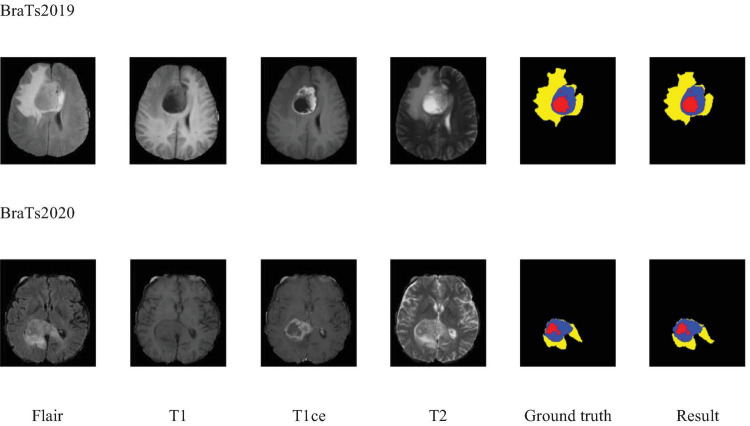
Visualization of the segmentation results of MAUNet. (Red, blue, and yellow indicate necrotic tumor core (NCR), enhanced tumor (ET), and peritumoral edema (ED), respectively).

**FIGURE 5:**
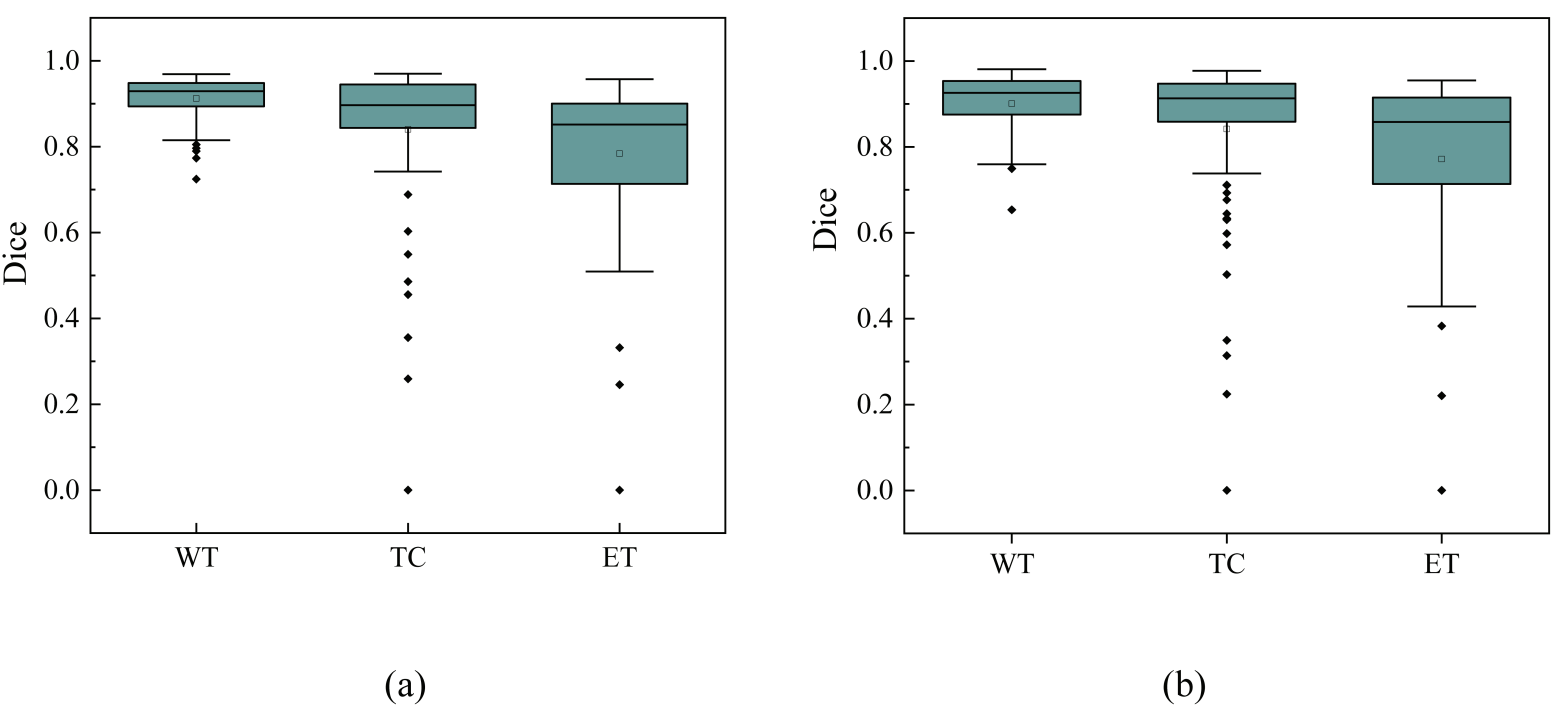
The box plot of MAUNet’s segmentation results, where **(a,b)** represent the segmentation outcomes for the BraTS2019 and BraTS2020 datasets, respectively.

Additionally, to visually demonstrate the segmentation outcomes, we processed the model outputs for visualization. Figure 4 illustrates that the segmentation findings indicate MAUNet’s capability to precisely identify lesion sites, highlighting the model’s potential for clinical applications. To further illustrate the segmentation results and provide a visual representation of their distribution, box plots (Boxplot) were utilized. Figure 5 presents box plots summarizing the segmentation performance of MAUNet on the BraTS2019 and BraTS2020 brain-tumor segmentation datasets. The median Dice coefficient of every box plot lies approximately between 0.80 and 1.00, demonstrating that MAUNet achieves high segmentation accuracy across the three tumor sub-regions. The low height of each box indicates that most observations are concentrated at high Dice values, confirming the model’s stable performance. There are also some outliers in the figure, but they are few in number, indicating that the model shows slight fluctuations on individual samples. Overall, Figure 5 shows that MAUNet performs well in the task of brain tumor segmentation, with high accuracy and stability.

**FIGURE 6:**
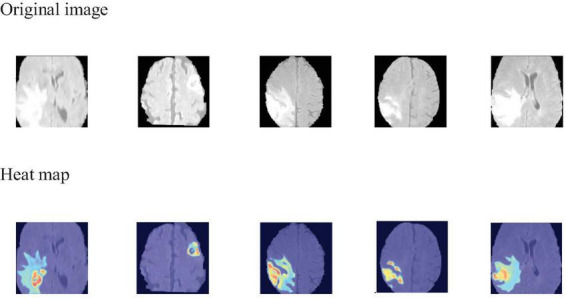
Grad-CAM visualization of MAUNet model segmentation results.

To visually identify the regions of interest on which the model focuses during the 3D brain tumor segmentation process, we employ Grad-CAM (Selvaraju et al., 2019) to generate visual explanations. As shown in Figure 6, the areas highlighted in red and yellow represent the regions that the model pays more attention to, while the blue and darker areas correspond to the regions that the model pays less attention to or receives no attention at all. This visualization technique aids medical professionals by highlighting critical regions within the scan, thereby directing their diagnostic focus and potentially assisting in the diagnostic process.

Ablation experiments were carried out on BraTS2019 to systematically evaluate each critical model component’s contribution. These studies systematically modified critical modules by either eliminating or substituting them, enabling observation of performance variations across configurations. This approach allowed us to quantify individual contributions to the segmentation accuracy, highlighting the functional necessity of distinct architectural elements. Detailed methodologies and quantitative outcomes of these component-level analyses are presented in later chapters, providing empirical evidence for the role of specific modules and their cumulative effects on system effectiveness.

### 4.6 Ablation experiments

To evaluate the efficacy of critical components within MAUNet, such as gate attention, SWDA, and CFC, ablation studies were conducted.

Firstly, each module was incorporated separately to evaluate its unique impact on model performance. Table 5 illustrates that the incorporation of a single module into the baseline model resulted in varied degrees of improvement in the overall Dice coefficient. Notably, method (3), which involves adding the SMWA module to the baseline, resulted in an average Dice improvement of 1.1%. However, it is worth noting that for the segmentation of the TC region, all methods enhanced with single modules performed worse than the baseline model. Similarly, methods (2) and (4) showed decreased performance for the ET region.

**TABLE 5:** Performance indicators of a single module.

Methods	Dice (%)			
	WT	TC	ET	Average
(1) Baseline	86.9	80.3	75.5	80.9
(2) Baseline + Gate	89.6	79.7	74.9	81.4
(3) Baseline + SMWA	89.9	79.7	76.5	82.0
(4) Baseline + CFC	89.4	79.6	75.4	81.5
(5) Baseline + Gate + CFC	89.8	81.2	75.6	82.3
(6) Baseline + Gate + SMWA	89.8	82.4	77.3	83.2
(7) Baseline + SMWA + CFC	90.0	81.6	77.1	82.9
(ours) MAUnet	91.2	84.0	78.4	84.5

### 4.7 Discussion

MAUNet, as previously detailed, achieved average Dice coefficients of 84.5 and 83.6% on the BraTS2019 and BraTS2020 datasets, respectively (refer to Figures 1, 2 and Table 2). To further validate the model’s effectiveness and better understand how its various components contribute, a series of ablation studies was conducted. These studies specifically examined the roles of the key components–Gate Attention, SWDA, and CFC. The results unequivocally affirm the necessity of these modules in improving the precision of brain tumor segmentation and demonstrate the advantages of their collaborative function.

The segmentation performance of MAUNet was benchmarked against the existing advanced techniques using the same dataset, and the comparison results are shown in Table 6.

**TABLE 6:** Comparison with existing methods.

Dataset	Method	Dice (%)			
		WT	TC	ET	Average
BraTS2019	Zhou, 2024	86.7	87.1	78.9	84.2
	Zhou and Zhu, 2023	86.5	87.0	79.4	84.3
	Li et al., 2022 and Sobhaninia et al., 2023	89.3	82.3	78.8	83.5
	Wang et al., 2021	90.0	81.9	78.9	83.6
	Peng and Sun, 2023	90	81	76	82.3
	Latif et al., 2021	87.4	75.8	74.1	79.1
	Akbar et al., 2022	88.5	81.0	74.9	81.5
	Huang et al., 2025	88.82	79.61	71.60	80.01
	Huang et al., 2025	90.81	76.08	68.57	78.49
	Ours	91.2	84.0	78.4	84.5
BraTS2020	Guan et al., 2022	85	69	67	73.7
	Wang et al., 2021	90.1	81.7	78.7	83.5
	Peng and Sun, 2023	90	80	76	82
	Ali et al., 2023	89.6	83.2	75.0	82.6
	Qamar et al., 2021	87.5	83.7	79.5	83.6
	Akbar et al., 2022	88.6	80.2	72.9	80.6
	Ours	90.1	84.2	77.2	83.8

On the BraTS2019 dataset, MAUNet’s Dice scores for the TC metric underperformed relative to models by Zhou (2024) and Zhou and Zhu (2023). Similarly, for the ET Dice score, MAUNet trailed Zhou (2024), Zhou and Zhu (2023), Li et al. (2022), Sobhaninia et al. (2023), and Wang et al. (2021). However, MAUNet outperformed these methods [cited in (Zhou and Zhu, 2023; Peng and Sun, 2023; Sobhaninia et al., 2023; Zhou, 2024; Wang et al., 2021; Akbar et al., 2022; Latif et al., 2021)] in terms of the average Dice score. On the BraTS2020 dataset, MAUNet achieved the highest TC Dice score, though its ET Dice score lagged behind the results reported by Wang et al. (2021) and Qamar et al. (2021). Nonetheless, MAUNet secured the top average Dice score across all metrics.

## 5 Conclusion

Brain tumors are a serious threat to human life, and computer-assisted diagnostic systems can really help lighten the load for doctors while also improving patient outcomes. In this study, we’re introducing MAUNet, a 3D MRI brain tumor segmentation model that uses mixed attention mechanisms. The SMDConv module, with its SMWA mechanism, allows for multi-dimensional feature modeling, which significantly boosts the model’s ability to represent complex structures. The CFC module, paired with dilated convolutions, captures multi-scale contextual information and fine-tunes the relationships between pixels and their context. On top of that, the gating mechanism selectively highlights the most diagnostically important features through refined fusion. We tested MAUNet on the BraTS 2019 and BraTS 2020 datasets, and the results are really promising. Compared to baseline models, MAUNet exhibits enhanced performance, achieving Dice scores of 91.2% (WT), 84.0% (TC), and 78.4% (ET) on BraTS2019, and 90.1% (WT), 84.2% (TC), and 77.2% (ET) on BraTS2020. Ablation studies confirm that each module plays a crucial role, and comparative analyses show that MAUNet outperforms existing methods. While this study focuses on brain tumor segmentation, the framework we’ve developed could potentially be applied more broadly. In future work, we will refine this architecture and systematically investigate more effective feature extraction techniques. (1) At each resolution level, the deepest SMDConv will be replaced by a lightweight Mamba–CapsResidual block to learn the part-whole relationship between tumor sub-regions (Zhang et al., 2025). (2) The existing CFC module will serve as a parallel context path, fusing capsule-activated holistic representations with CFC-calibrated pixel-level features via a cross-attention mechanism (Liu et al., 2025).

## Data Availability

Publicly available datasets were analyzed in this study. This data can be found here: The BraTS2019 and BraTS2020 datasets are publicly available for research purposes. BraTS2019 data set from (https://www.med.upenn.edu/cbica/brats2019/data.html). BraTS2020 data set from (https://www.med.upenn.edu/cbica/brats2020/data.html).
